# Comparative prebiotic activity of mixtures of cereal grain polysaccharides

**DOI:** 10.1186/s13568-019-0925-z

**Published:** 2019-12-21

**Authors:** Suzanne Harris, Andrea Monteagudo-Mera, Ondrej Kosik, Dimitris Charalampopoulos, Peter Shewry, Alison Lovegrove

**Affiliations:** 1 Department of Food and Nutritional Sciences, University of Reading, Whiteknights, PO Box 226, Reading, RG6 6AP UK; 2 Department of Plant Science, Rothamsted Research, Harpenden, AL5 2JQ Hertfordshire UK

**Keywords:** Prebiotic, Batch culture, Fluorescence in situ hybridisation (FISH), Short chain fatty acids (SCFA), β-Glucan, Arabinoxylan

## Abstract

The main components of the non-starch polysaccharide (NSP) fraction of wheat flour are arabinoxylan (AX) and β-glucan. These are also present in other cereal grains, but their proportions vary with AX being the major component in wheat and rye and β-glucan in barley and oats. Therefore, it was hypothesised that these NSPs could act synergistically when fermented in vitro at the ratios present in the major foods consumed, resulting in increased prebiotic activity. AX and β-glucan were therefore tested in in vitro fermentation studies to assess their prebiotic activity when used individually and/or in different ratios. Short-chain fatty-acids (SCFAs) produced from in vitro fermentation were measured using HPLC and bacterial populations were measured using flow cytometry with fluorescence in situ hybridisation (Flow-FISH). Fermentation of AX alone resulted in a significant bifidogenic activity and increased concentrations of SCFAs, mainly acetate, after 8–24 h of fermentation, however β-glucan alone did not show prebiotic activity. The greatest prebiotic activity, based on concentration of total SCFAs and increases in total bacteria as well as beneficial *Bifidobacterium* and *Clostridium coccoides/Eubacterium* groups, was observed when AX and β-glucan were combined at a 3:1 ratio, which corresponds to their ratios in wheat flour which is major source of cereal fibre in the diet. This indicates that the population of bacteria in the human GI tract may be modulated by the composition of the fibre in the diet, to maximise the prebiotic potential.

## Introduction

Wheat is most important cereal in terms of global consumption, being the staple food crop in temperate countries and increasingly replacing traditional crops in sub-Saharan Africa and Asia. Wheat is usually consumed after processing into two types of food: as bread and other baked goods and as noodles or pasta. In most cases these are produced from white flour which is made from the endosperm and contains around 2–3% total dietary fibre (TDF) compared with wholemeal which also contains the outer bran layers and contains about 11.5 to 15.5% TDF (Shewry and Hey 2015). Nevertheless, white bread contributes about 10% of the TDF in the adult UK diet (Steer et al. 2008), and correspondingly more in regions such as North Africa and Central Asia where wheat can account for between 50 and 70% of the total energy intake. Hence, the biological activity of wheat flour fibre is of significant interest, including their properties as a prebiotic (which can be defined as ‘non-digestible (by the host) food ingredients that have a beneficial effect through their selective metabolism in the intestinal tract’ (Gibson et al. 2017).

The major dietary fibre (DF) components in wheat flour are cell wall polysaccharides, fructans, resistant starch and the arabinogalactan peptide. Fructans account for 1–2% of white flour (Haskå et al. 2008) and have well-established prebiotic properties, while the level of resistant starch is low (< 0.1%) compared to non-starch polysaccharides (Siljeström and Asp 1985). The cell wall polysaccharides of wheat flour comprise two major components, about 70% arabinoxylan (AX) and 20% (1 → 3,1 → 4)-β-d-glucan (β-glucan), with about 2% cellulose ((1 → 4)-β-d-glucan) and 7% glucomannan (Mares and Stone 1973). AX comprises a backbone of β-d-xylopyranosyl residues linked through (1 → 4) glycosidic linkages with some residues being substituted with α-l-arabinofuranosyl residues at either position 3 or positions 2 and 3 (Fincher and Stone 1974). Some arabinofuranosyl residues at position 3 of the xylan residues may themselves be substituted with ferulic acid at the 5 position which allows the formation of cross-links, by oxidation of ferulate present on adjacent AX chains to give dehydrodimers (diferulates). AX occurs in water-soluble and insoluble forms, which may differ in their molecular weight, degree of substitution, and extent of diferulate cross-linking. β-glucan has a simpler structure, comprising only glucose residues joined by (1 → 3) and (1 → 4) linkages. Single (1 → 3) linkages are usually separated by two or three (1 → 4) linkages but longer stretches of (1 → 4) linked glucan of up to 14 units have been reported for wheat bran β-glucan (Li et al. 2006). Such regions are sometimes referred to as “cellulose-like”, as cellulose is (1 → 4)-β-d-glucan without any (1 → 3) linkages. β-Glucan occurs in soluble and insoluble forms, which may differ in their size and distribution of (1 → 3) and (1 → 4) linkages (Johansson et al. 2004).

Several previous studies of the prebiotic effects of AX from wheat and β-glucan from barley have been reported (Hughes et al. 2007, 2008; Wang et al. 2016). However, should be noted that these components are not consumed singly in human diets but as mixtures in complex foods. In particular, AX and β-glucan are most widely consumed in a ratio of about 3:1 in bread and other wheat products and it is therefore possible that the human colonic microflora has adapted to provide more efficient fermentation of this ratio. We have therefore compared the fermentation of AX and β-glucan as single compounds with mixtures at ratios of 1:3, 1:1 and 3:1.

## Materials and methods

### Materials

Wheat arabinoxylan (P-WAXYM) and barley β-glucan preparations (P-BGBH) were purchased from Megazyme (Bray, co. Wicklow, Ireland).

### *In*-*vitro* fermentation

100 mL sterile batch fermentation vessels (50 mL working volume) were aseptically filled with 45 mL of sterile basal medium and sparged with O_2_-free N_2_ overnight to establish anaerobic conditions. The medium contained per litre: 2 g of peptone water (Oxoid Ltd., Basingstoke, United Kingdom), 2 g of yeast extract (Oxoid), 0.1 g of NaCl, 0.04 g of K_2_HPO_4_, 0.01 g of MgSO_4_·7H_2_O, 0.01 g of CaCl_2_·6H_2_O, 2 g of NaHCO_3_, 0.005 g of hemein (Sigma), 0.5 g of l-cysteine HCl (Sigma), 0.5 g of bile salts (Oxoid), 2 mL of Tween 80, 10 µL of vitamin K (Sigma). Polysaccharide samples were added (1% w/v) to the basal medium. Each vessel was inoculated with 10% (v/v) of faecal slurry, which was prepared by homogenizing fresh human faeces (10%, w/w) in phosphate-buffered saline (PBS; 8 g/L NaCl, 0.2 g/L KCl, 1.15 g/L Na_2_HPO_4_, and 0.2 g/L KH_2_HPO_4_), pH 7.3 (Oxoid), using a stomacher (Stomacher 400, Seward). Three individual faecal donors were used per experiment and samples were not pooled, donors were two female and one male, between 23 and 59 years of age and on a normal diet without any special dietary requirements and that had not taken antibiotics, prebiotic or probiotics in the previous 3 months. Vessels were incubated at 37 °C with a water jacket for up to 48 h and the pH was controlled between 6.7 and 6.9 with 0.5 M HCl and 0.5 M NaOH using an automated pH controller (Fermac 260, Electrolab, Tewkesbury, UK). Samples were collected at 0, 4, 8, 24 h for analysis.

### SCFA analysis using HPLC

Samples were centrifuged at 13,000×*g* for 5 min to remove particulate matter and filtered using 0.2 μM nitrocellulose filter. 20 μL was injected on to a Phenomenex Rezex ROA Organic Acid H^+^ (8%) HPLC column (Watford, UK) at 50 °C on a Shimadzu HPLC with 0.0025 M H_2_SO_4_ eluent at a flow rate of 0.6 mL/min. SCFA (lactate, formate, acetate, propionate and butyrate) were quantified using standard calibration curves from 1 to 100 mM.

### Enumeration of bacteria by flow-FISH

Samples were centrifuged at 10,000×*g* for 3 min and supernatant discarded. Pelleted sample was fixed for 4 h at 4 °C with 4% (w/v) filtered paraformaldehyde (pH 7.2) in a ratio of 1:3 (v/v). Samples were washed twice with filtered PBS and resuspended in 600 μL of a mixture of PBS/ethanol (1:1, v/v) and then stored at − 20 °C for up to 3 months. Hybridisation was carried out as described in Rycroft et al. (2001a, b) using genus and group specific 16S rRNA-targeted oligonucleotide probes (MWG Biotech, Ebersberg, Germany).

The sample probes used were Bif164 (Langendijk et al. 1995), Bac303 (Manz et al. 1996), Lab158 (Harmsen et al. 2000), Ato291^3^, Prop853 (Walker et al. 2005), Erec482 (Franks et al. 1998), Rrec584 (Walker et al. 2005), Fprau655 (Hold et al. 2003), Chis150 (Franks et al. 1998), shown in Additional file 1: Table S1. Samples were screened using a flow cytometer (Accuri C6, BD Biosciences, USA) with Accuri CFlow software.

### Statistical analysis

Statistical analyses were performed using SPSS for Windows, version 21. Univariate analysis of variance and Tukey’s post hoc test was used to determine significant changes between treatments in the microbiota populations and SCFA concentrations. Differences were considered significant when P < 0.05.

## Results

### Production of SCFA

**Fig. 1:**
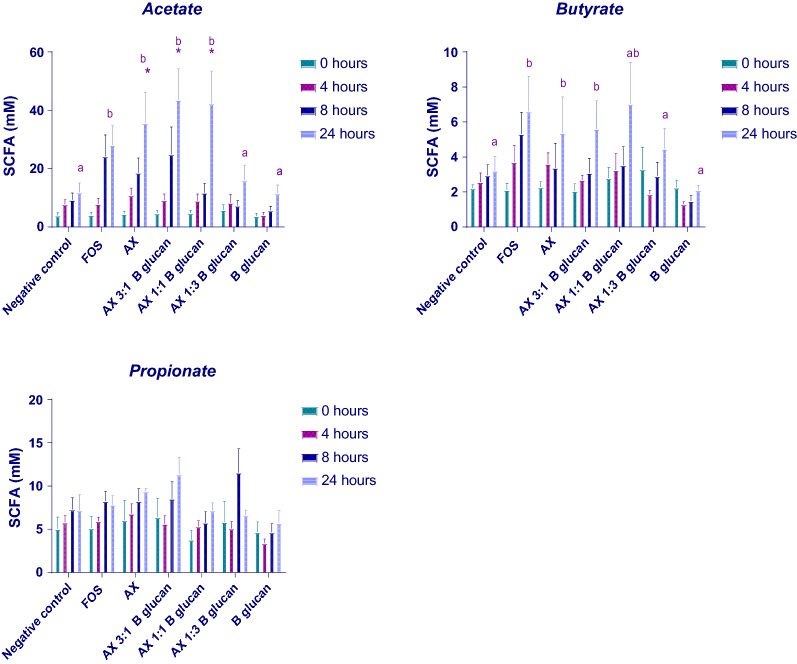
SCFA analysed by HPLC in batch cultures containing different substrates: Error bars indicate SEM (n = 3). Significant differences between substrates at the same time point are indicated with *P < 0.05. One-way ANOVA with Tukey’s post hoc tests were used for statistical analysis

Figure 1 and Additional file 1: Table S2 show the concentrations of SCFAs and lactate in samples incubated with: AX alone, AX: β-glucan (3:1, w/w), AX: β-glucan (1:1, w/w), AX: β-glucan (1:3, w/w), β-glucan alone and FOS after 24 h’ fermentation. Fermentation of the samples containing AX, AX: β-glucan (3:1), AX: β-glucan (1:1), and FOS had significantly higher concentrations of total SCFAs (P > 0.95) compared to the negative control, mainly due to production of acetate. The greatest increase in acetate concentration was observed with AX and AX: β-glucan (3:1) from 8 to 24 h, and with AX: β-glucan (1:1) at 8 h. By contrast, the acetate concentrations were not significantly increased compared to the control sample with AX: β-glucan (1:3) or β-glucan alone. While the mean butyrate concentration was increased after 24 h fermentation with FOS, AX, AX: β-glucan (3:1), and AX: β-glucan (1:1), these increases were not statistically significant due to large variation between donors.

Table 1 shows the percentages of individual SCFAs produced after 24 h fermentation. The proportions of SCFAs differ between substrates, with those comprising high proportions of AX producing greater proportions of acetate and those containing greater levels of β-glucan producing greater proportions of propionate. The proportion of butyrate produced did not differ.

**Table 1:** % SCFA produced in in vitro colonic fermentation vessels at 24 h containing AX and β-glucan alone and combined in different ratios

	SCFA (%)		
	Acetate	Propionate	Butyrate
No treatment	47.2 (± 13.5)	37.0 (± 7.8)	15.7 (± 4.1)
FOS	70.0 (± 8.0)	15.4 (± 4.3)	14.6 (± 4.6)
AX	73.5 (± 16.1)	15.4 (± 3.6)	11.1 (± 4.3)
AX 3:1 β-glucan	74.9 (± 10.6)	15.5 (± 4.7)	9.6 (± 2.8)
AX 1:1 β-glucan	72.0 (± 25.3)	12.0 (± 3.7)	15.9 (± 5.4)
AX 1:3 β-glucan	63.0 (± 16.5)	19.3 (± 5.7)	17.7 (± 4.5)
β-Glucan	45.3 (± 10.3)	44.9 (± 17.5)	9.7 (± 1.3)

FOS is the positive control, and no added polysaccharide (no treatment) is the negative control, n = 3

### Bacterial populations

**Fig. 2:**
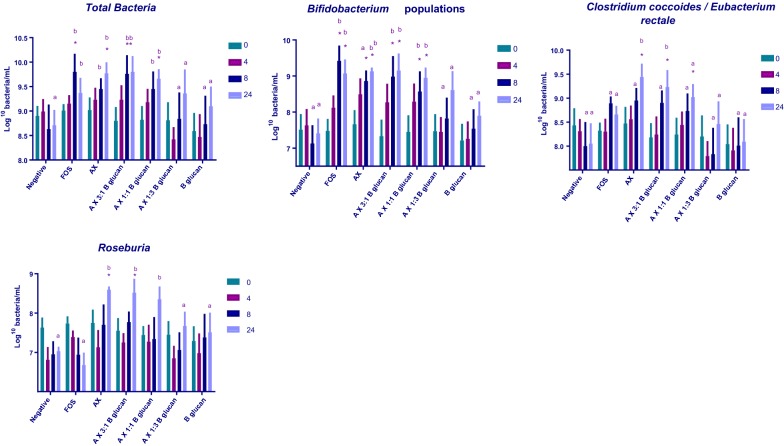
Bacterial populations analysed by Flow-FISH in batch cultures containing different substrates: Error bars indicate SEM (n = 3). Significant differences between substrates at the same time point are indicated with *P < 0.05. One-way ANOVA with Tukey’s post hoc tests were used for statistical analysis

The populations of the dominant types of human colonic bacteria after in vitro fermentation with AX alone, AX: β-glucan (3:1), AX: β-glucan (1:1), AX: β-glucan (1:3), β-glucan alone and FOS are shown in Additional file 1: Table S3, while the populations which changed significantly compared with the negative control (Total bacteria, *Bifidobacterium*, *Clostridium coccoides/Eubacterium rectale* and *Roseburia*) are shown in Fig. 2. AX, AX: β-glucan (3:1) and AX: β-glucan (1:1) had significant (P < 0.05) bifidogenic effects between 8 and 24 h while the same three substrates gave significant increases *Clostridium coccoides/Eubacteium rectale* compared to the negative control (P < 0.05) after 24 h. The populations of *Roseburia* populations also increased significantly compared to the negative control (P < 0.05) with fermentation of AX and AX: β-glucan (3:1) after 24 h. Total bacterial populations included genera that were not specifically targeted with fluorescent probes, AX and AX: β-glucan (1:1) showed significant (P < 0.05) increases in total bacteria after 24 h, however AX: β-glucan (3:1) demonstrated earlier increases between 8 and 24 h.

## Discussion

This study aimed to determine the prebiotic activity of AX and β-glucan in combination and in different ratios in order to determine the optimal ratio of AX: β-glucan for the highest prebiotic activity.

A prebiotic is defined as a substrate that is selectively utilized by host microorganisms conferring a health benefit (Gibson et al. 2017) and the two major types of dietary fibre present in cereal grains have been shown to have prebiotic activity: wheat AX (Grootaert et al. 2009; Hughes et al. 2007; Van Craeyveld et al. 2008) and barley β-glucan (Hughes et al. 2008; Wang et al. 2016).

Short-chain fatty acids (SCFA) are volatile fatty acids consisting of a straight-chain aliphatic tail of fewer than six carbon atoms and are produced by oligosaccharide fermentation concomitant with increase in beneficial bacteria including *Bifidobacterium.* Their production is therefore used to measure prebiotic activity together with beneficial changes in the microbiota. The principal SCFAs produced are acetate, propionate and butyrate (comprising 95% of the total) (Cummings et al. 1987) and are metabolized by the colonic epithelium (butyrate), liver (propionate) and muscle (acetate) (Cummings and Macfarlane 1997). Relatively little is known about the role of formate in the gut, however it has been linked to methanogenesis and appears to be elevated in inflammatory conditions (Bereswill et al. 2011; Vanderhaeghen et al. 2015). SCFAs have been shown to provide multiple beneficial effects for the host, for example, providing dietary energy and suppressing the growth of pathogens by decreasing the pH of the intestinal lumen (Blaut 2002). The concentration of SCFAs in this study was used to measure the rate of fermentation of the substrates, with significant increases particularly apparent in the predominant SCFA, acetate, which is often utilised to produce other SCFAs, butyrate and propionate.

Acetate was the highest contributor of total SCFA for all samples. The addition of larger amounts of AX appears to drive the concentration towards greater acetate production (Table 1), whereas greater β-glucan appears to favour propionate production. The highest proportion of propionate is seen with the β-glucan sample alone at 44.9% (although this is at a significantly lower concentration than acetate). Previous studies have shown fermentation of β-glucan to favour production of propionate and fermentation of AX to encourage acetate and butyrate (Hughes et al. 2007, 2008) and these results corroborate these previous findings.

The bacterial genus *Bifidobacterium* is most often targeted by prebiotics, as it is associated with multiple health benefits, including reducing the proliferation of colorectal cancer and the concentration of circulating cholesterol (Singh 1997; Zanotti et al. 2015). Increases in *Bifidobacterium* populations were observed with the samples AX, AX: β-glucan (3:1), AX: β-glucan (1:1) and FOS (the positive control). Only FOS and the samples containing AX at a concentration of least 50% showed an increase in bifidobacteria, demonstrating a bifidogenic effect for AX but not β-glucan and supporting previous studies which showed that fermentation of oat and barley β-glucans had no effect on *Bifidobacterium* populations, whilst AX fermentation resulted in increases in *Bifidobacterium* populations (Hughes et al. 2007, 2008; Kim and White 2009). Concomitant increases in acetate were observed in all samples, which is consistent with the established role of bifidobacteria in acetate production (Bindels et al. 2015; Fukuda et al. 2011).

The structures of fermentable carbohydrates, including the degree of polymerisation (DP) and molecular weight, have previously been shown to affect the rate of fermentation (Hughes et al. 2007) and FOS is thought to be rapidly fermented due to its low DP (Stewart et al. 2008). In this study, the molecular masses of the AX (323 kDa) and β-glucan (491 kDa) were much greater than that of FOS (DP 2–8). The longer polysaccharides in AX and β-glucan contain fewer non-reducing ends per unit mass than FOS, providing less substrate for hydrolysis by bacterial enzymes. However, a slower rate of fermentation could be beneficial slow-fermenting prebiotic may be able to reach the more distal regions of the colon, where the fermentable carbohydrate levels are much lower, and fermentation of proteins is more prevalent, and therefore have a greater impact on colonic health (Govers et al. 1999; Grootaert et al. 2009). The increase in total bacteria in samples with greater proportions of AX [AX, AX: β-glucan (3:1), AX: β-glucan (1:1)] were more sustained than with FOS, peaking at 24 h compared to 8 h with FOS and demonstrating greater persistence. The AX: β-glucan (3:1) sample showed significant increases from the negative control after 8 h and continued until 24 h, therefore demonstrating the most sustained fermentation.

The increases in bifidobacteria were sustained between 8 and 24 h with AX and AX: β-glucan (3:1). The populations of *Clostridium coccoides/Eubacterium rectales* were also highest in both the AX and AX: β-glucan (3:1) samples at 24 h. The increased populations of these bacterial groups at later fermentation times therefore demonstrates longer fermentation of samples containing higher levels of AX.

Similar increases in *Bifidobacterium, Clostridium coccoides/Eubacterium rectales* and *Roseburia* populations resulted from fermentation of AX alone and a 3:1 combination of AX and β-glucan, as well as similar production of SCFAs. However, samples with AX: β-glucan (1:3) and β-glucan singly did not cause increases in beneficial bacterial groups of SCFAs It has been proposed that three colonic *Bacteroides* species: *Bacteroides thetaiotaiomicron*, *Bacteroides distasonis* and *Bacteroides fragilis* are responsible for the majority of β-d-(1 → 3)-glucanase activity required for β-glucan hydrolysis (Salyers et al. 1997), however no significant increases were observed in the *Bacteroides* group with any combination of substrates. These results may be explained by a low prevalence of these particular *Bacteroides* species in the samples, and therefore a low level of β-glucan fermentation.

Butyrate is produced by a range of bacteria including the *Clostridium, Roseburia* and *Eubacterium* genera (Barcenilla et al. 2000; Gibson 1999; Pryde et al. 2002). Despite the increase in these butyrogenic bacterial populations with AX and AX and β-glucan (3:1), and a large rise in mean butyrate concentration, there were no significant concomitant increase in butyrate. However, closer inspection of the data shows that donors 1 and 3 responded to fermentation of all samples containing AX with production of butyrate, whilst butyrate production with donor 2 only responded to fermentation of FOS. Hence, the failure to observe significant increases in butyrate resulted in differences between individual donors.

The greatest increases in total bacterial numbers were observed with both the AX alone and the AX: β-glucan (3:1) sample, while no increase in bacterial numbers occurred when β-glucan alone was used, suggesting that AX is more readily used as a substrate for growth by bacteria than β-glucan. As the ratio of AX: β-glucan is greater in wheat (3:1) than barley (1:3), these data supports a previous study which showed that a wheat-based diet in pigs resulted in a greater increase in gut bacteria than a barley-based diet (Garry et al. 2007).

Thus, it appears that AX is readily used as a substrate for fermentation, at a greater rate than β-glucan, as previously described (Hughes et al. 2007, 2008) and that total bacterial numbers, and the *Bifidobacterium*, *Roseburia* and *Clostridium coccoides*–*Eubacterium rectale* bacterial groups show preferential growth with AX as a substrate. However, AX supplemented with β-glucan in a ratio of 3:1 showed similar increases in these bacterial populations and slightly greater SCFA concentrations as well as greater increases in total bacteria compared to AX alone, indicating that replacing 25% AX as a fermentable substrate with 25% β-glucan can potentially increase prebiotic activity. Any greater replacement of AX was shown to result in decreases in bacterial populations and SCFA. Wheat has been widely consumed by humankind for thousands of years, we therefore hypothesised that the human gut microbiota may have co-evolved to ferment the ratio of DF polysaccharides present in wheat more efficiently than other ratios of polysaccharides. The results reported here provide support for this hypothesis, as beneficial bacteria were shown to preferentially ferment AX compared to β-glucan with the preferred ratio of AX: β-glucan being that present in wheat (3:1).

## Supplementary information

**Additional file 1.** Tables containing information on fluorescence in situ hybridisation probes used in the study. Tables containing data points for SCFA concentrations and bacterial populations after fermentation experiments.

## Data Availability

Original data sets will not be shared as it is not a funder requirement.
