# Supplementary material for: Comparative prebiotic activity of mixtures of cereal grain polysaccharides
Source: AMB Express. 2019 Dec 21;9:203. doi: 10.1186/s13568-019-0925-z (PMC6925609; doi:10.1186/s13568-019-0925-z)
Supplement: Supplementary file 1 — Additional file 1. Tables containing information on fluorescence in situ hybridisation probes used in the study. Tables containing data points for SCFA concentrations and bacterial populations after fermentation experiments. [file 13568_2019_925_MOESM1_ESM.pdf]

AMB Express

Comparative prebiotic activity of mixtures of cereal grain polysaccharides

Suzanne Harris[1,2], Andrea Monteagudo-Mera[1], Ondrej Kosik[2], Dimitris Charalampopoulos[1], Peter Shewry[1,2], Alison Lovegrove[2]

[1]Department of Food and Nutritional Sciences, University of Reading, Whiteknights, PO Box 226, Reading RG6 6AP, UK

[2]Department of Plant Science, Rothamsted Research, Harpenden, AL5 2JQ, Hertfordshire, UK

Corresponding author: Suzanne.harris@quadram.ac.uk, +44 (0)1603 255000

Table S1: 16S rRNA-targeted oligonucleotide probes

Probe Name	Sequence (5' to 3')	Fluorescence	Targeted Bacteria	
Non Eub	ACTCCTACGGGAGGCAGC	Alexa-488	None	Wallner et al., 1993)
Eub338I*	GCTGCCTCCCGTAGGAGT	Alexa-488	All	Daims et al., 1999)
Eub338II*	GCAGCCACCCGTAGGTGT	Alexa-488	All	
Eub338III*	GCTGCCACCCGTAGGTGT	Alexa-488	All	
Non Eub	ACTCCTACGGGAGGCAGC	Alexa-647	None	Wallner et al., 1993)
Eub338I	GCTGCCTCCCGTAGGAGT	Alexa-647	All	Daims et al., 1999)
Eub338II	GCAGCCACCCGTAGGTGT	Alexa-647	All	
Eub338III	GCTGCCACCCGTAGGTGT	Alexa-647	All	
Bif164	CATCCGGCATTACCACCC	Alexa-647	*Bifidobacterium*	(Langendijk et al., 1995
Lab158	GGTATTAGCAYCTGTTTCCA	Alexa-647	*Lactobacillus* and *Enterococcus*	H.J.M. Harmsen et al., 1999)
Bac303	CCAATGTGGGGGACCTT	Alexa-647	*Bacteroides*	(Manz et al., 1996)
Erec482	GCTTCTTAGTCARGTACCG	Alexa-647	*Eubacterium rectale/Clostridium coccoides* cluster	(Franks et al., 1998)
Rrec584	TCAGACTTGCCGYACCGC	Alexa-647	*Roseburia*	(Walker et al., 2005)
Ato291	GGTCGGTCTCTCAACCC	Alexa-647	*Atopobium*	(H. J.M. Harmsen et al., 2000)
Prop853	ATTGCGTTAACTCCGGCAC	Alexa-647	*Clostridium cluster IX*	(Walker et al., 2005)
Fprau655	CGCCTACCTCTGCACTAC	Alexa-647	Faecalibacterium prausnitzii	(Devereux et al., 1992
DSV687	TACGGATTTCACTCCT	Alexa-647	*Desulfovibrionales*	(Hold et al., 2003)
Chis150	TTATGCGGTATTAATCTYCCTTT	Alexa-647	*Clostridium histolyticum*	Franks et al., 1998)

Table S2: **SCFA and lactate concentration in batch cultures at 0, 4, 8 and 24 hours' fermentation comparing no treatment, FOS and different ratios of AX and β-glucan.** Negative control is no added carbohydrate and positive control is FOS. One-way ANVOA and post hoc tukey test were used. SEM is shown in italics. Significant interaction between sample and its time 0 are denoted with letters. Significant differences between treatments are denoted with* ($p < 0.05$) and shown in bold. Formate is included in total concentration but not shown.

	Time	Lactate (μM)	Acetate (μM)	Propionate (μM)	Butyrate (μM)	Total (μM)
No treatment	0	0.93a (0.40)	3.88a (0.92)	5.05a (1.37)	1.85a (0.19)	16.27a (0.26)
	4	4.79a (1.41)	7.77a (1.56)	5.79a (0.81)	2.28a (0.53)	25.85a (1.10)
	8	3.57a (1.50)	9.25a (2.38)	7.28a (1.37)	2.72a (0.61)	29.44b (3.91)
	24	1.72a (0.50)	11.83a (3.23)	7.17a (1.80)	3.27a (0.84)	29.26a (2.35)
FOS	0	3.75a (2.53)	4.05a (0.96)	5.13a (1.39)	2.04a (0.38)	18.72a (3.32)
	4	6.17a (2.76)	7.86a (1.98)	5.94a (0.46)	4.03a (0.96)	29.03a (2.40)
	8	21.62a (6.52)	**24.25b*** (7.28)	8.24a (1.11)	5.59a (1.25)	**78.80c*** (2.11)
	24	7.43a (3.96)	**28.16b*** (6.49)	7.82a (1.05)	6.60a (1.86)	**62.42b*** (2.83)
AX	0	5.01a (4.03)	4.46a (0.94)	6.04a (2.29)	1.47a (0.30)	21.74a (2.63)
	4	9.84a (4.93)	10.89a (2.42)	5.65a (1.12)	3.23a (0.63)	37.29b (3.93)
	8	6.28a (2.59)	**18.61b*** (5.09)	8.52a (1.43)	3.64a (1.40)	**47.57c*** (4.35)
	24	2.12a (0.62)	**35.59b*** (10.56)	11.34b (0.32)	5.96a (2.06)	**66.84b*** (6.33)
AX 3:1 B glucan	0	1.96a (0.94)	4.70a (0.99)	6.40a (2.17)	1.81a (0.41)	20.15a (1.27)
	4	10.41a (4.73)	9.12a (2.16)	5.65a (0.92)	2.21a (0.27)	34.32b (2.27)
	8	11.43a (5.63)	**24.94b*** (9.35)	8.52a (1.97)	3.34a (0.82)	**61.14c*** (4.18)
	24	3.97a (0.94)	**43.63b*** (10.68)	11.35b (1.97)	6.24a (1.61)	**75.90b*** (6.88)
AX 1:1 B glucan	0	5.18a (5.25)	4.66a (1.02)	3.80a (1.10)	2.05a (0.61)	19.05a (1.76)
	4	6.50a (4.62)	8.99a (2.31)	5.33a (0.66)	2.95a (0.95)	27.20a (1.64)
	8	7.15a (5.85)	11.71a (3.17)	5.77a (1.23)	2.67a (1.05)	**35.35b*** (2.75)
	24	1.24a (0.60)	**42.36b*** (11.15)	7.17a (0.88)	6.64a (2.37)	**67.82b*** (5.79)
AX 1:3 B glucan	0	2.67a (1.24)	5.84a (1.73)	5.84a (2.39)	2.94a (1.28)	24.16a (1.22)
	4	5.47a (3.40)	8.29a (2.83)	5.10a (0.79)	1.67a (0.24)	26.04a (1.96)
	8	5.42a (3.41)	7.25a (1.79)	11.56b (2.74)	2.96a (0.78)	36.58b (2.53)
	24	1.68a (0.48)	15.96a (5.26)	6.63a (0.58)	4.74a (1.15)	36.11a (3.57)
B glucan	0	0.87a (0.41)	3.75a (0.89)	4.68a (1.17)	1.70a (0.44)	15.54a (1.16)
	4	2.78a (1.22)	4.09a (0.86)	3.38a (0.49)	1.03a (0.15)	14.57a (1.01)
	8	2.78a (1.02)	5.65a (1.38)	4.67a (0.99)	1.33a (0.33)	18.12a (1.12)
	24	2.72a (0.97)	11.49b (2.90)	5.71a (1.42)	1.69a (0.29)	26.97b (2.56)

Table S3: Bacterial enumeration (mean $\log_{10}$ bacterial numbers / mL) of samples taken from static batch cultures after 0, 4, 8 and 24 hours' fermentation comparing no substrate, FOS and different ratios of AX and β-glucan Negative control is no added carbohydrate and positive control is FOS. Values are mean $\log_{10}$ bacterial numbers/mL found using flow FISH. One-way ANVOA and post hoc tukey test were used. SEM is shown in italics. Significant interaction between sample and its time 0 are denoted with letters ($p < 0.05$). Significant differences between treatments are denoted with * and shown in bold.

	Time (h)	*Bifidobacterium* genus	*Lactobacillus Enterococcus* group	*Bacteroides-Prevotella* group	*Clostridium coccoides-Eubacterium rectale* group	*Roseburia*	*Atopobium* cluster	*Clostridium* cluster IX	*Faecalibacterium prausnitzii group*	*Desulfovibrionales*	*Clostridium-cluster I and II*	Total
	0	7.54a (0.32)	7.32a (0.10)	7.66a (0.14)	8.43a (0.20)	7.63a (0.09)	7.41a (0.06)	8.04a (0.02)	8.30a (0.13)	7.79a (0.20)	7.14a (0.34)	9.00a (0.14)
	4	7.76a (0.39)	6.91a (0.12)	7.92a (0.11)	8.31a (0.10)	6.81a (0.16)	7.04a (0.26)	7.90a (0.19)	8.33a (0.10)	8.05a (0.10)	7.28a (0.11)	8.99a (0.10)
	8	7.27a (0.51)	6.83a (0.21)	7.49a (0.56)	8.00a (0.40)	6.95a (0.16)	7.14a (0.21)	7.60a (0.49)	7.89a (0.45)	7.57a (0.38)	7.03a (0.31)	8.63a (0.41)
Negative	24	7.56a (0.37)	7.18a (0.02)	7.27a (0.35)	8.05a (0.29)	7.20ab (0.06)	7.51a (0.14)	7.66a (0.14)	7.65a (0.26)	7.73a (0.12)	7.34a (0.10)	8.74a (0.17)
	0	7.48a (0.16)	7.39a (0.09)	7.86a (0.08)	8.32a (0.04)	7.73a (0.05)	7.60a (0.08)	8.26a (0.06)	8.34a (0.05)	7.82a (0.01)	7.30a (0.20)	9.01a (0.02)
	4	8.12a (0.17)	7.48a (0.20)	7.85a (0.14)	8.30a (0.11)	7.39a (0.03)	8.06a (0.07)	8.26a (0.08)	8.51a (0.10)	8.25a (0.03)	7.63a (0.10)	9.15a (0.04)
	8	**9.42b*** (0.27)	6.97a (0.11)	8.29a (0.26)	8.89a (0.03)	6.94a (0.29)	8.31a (0.35)	8.52a (0.25)	8.78a (0.22)	8.47a (0.26)	7.95a (0.15)	**9.80b*** (0.21)
FOS	24	**9.07b*** (0.23)	7.14a (0.13)	7.71a 0.18)	8.66a (0.04)	6.68a (0.15)	7.91a (0.26)	7.47a (0.13)	6.84a (0.32)	7.68a (0.25)	7.13a (0.36)	9.37b (0.14)
	0	7.66a (0.23)	7.42a (0.06)	7.84a (0.17)	8.47a (0.19)	7.75a (0.17)	7.43a (0.09)	8.07a (0.11)	8.28a (0.09)	7.91a (0.15)	7.20a (0.16)	9.02a (0.10)
	4	8.49a (0.37)	6.65a (0.03)	7.92a (0.04)	8.56a (0.15)	7.13a (0.36)	6.73a (0.35)	7.60a (0.42)	8.24a (0.04)	7.93a (0.02)	6.91a (0.18)	9.23a (0.11)
	8	**8.86ab*** (0.15)	6.60a (0.23)	7.62a (0.49)	8.95a (0.12)	7.70a (0.52)	6.78a (0.19)	7.32a (0.61)	7.66a (0.70)	6.80a (0.59)	6.51a (0.01)	9.45b (0.09)
AX	24	**9.13b*** (0.28)	8.04a (0.13)	8.50a (0.11)	**9.44b*** (0.14)	**8.59b*** (0.06)	7.93a (0.05)	8.50a (0.10)	7.94a (0.28)	8.12a (0.06)	8.01a (0.19)	**9.77b*** (0.01)

	Time (h)	*Bifidobacterium* genus	*Lactobacillus Enterococcus* group	*Bacteroides- Prevotella* group	*Clostridium coccoides- Eubacterium rectale* group	*Roseburia*	*Atopobium* cluster	*Clostridium* cluster IX	*Faecalibacterium prausnitzii group*	*Desulfovibrionales*	*Clostridium-cluster I and II*	Total
AX 3:1 B glucan	0	7.33a (0.33)	7.17a (0.11)	7.55a (0.19)	8.18a (0.14)	7.55a (0.15)	7.25a (0.04)	7.80a (0.02)	8.12a (0.11)	7.68a (0.23)	7.02a (0.27)	8.80a (0.12)
	4	8.27a (0.41)	6.77a (0.26)	8.24a (0.25)	8.24a (0.22)	7.25a (0.08)	7.72a (0.27)	8.28a (0.08)	8.42a (0.04)	8.11a (0.02)	7.17a (0.14)	9.23a (0.13)
	8	**8.98b*** (0.52)	7.16a (0.39)	8.56a (0.27)	8.90a (0.10)	7.77a (0.10)	8.06a (0.22)	8.55a (0.13)	8.68a (0.05)	8.25a (0.11)	7.50a (0.16)	**9.76b*** (0.23)
	24	**9.15b*** (0.35)	7.91a (0.27)	8.37a (0.17)	**9.23b*** (0.19)	**8.52b*** (0.05)	7.78a (0.65)	8.19a (0.38)	8.17a (0.17)	7.79a (0.46)	7.54a (0.43)	**9.80b*** (0.16)
AX 1:1 β-glucan	0	7.45a (0.33)	7.17a (0.08)	7.50a (0.09)	8.24a (0.19)	7.44a (0.07)	7.25a (0.04)	7.87a (0.04)	8.11a (0.12)	7.65a (0.20)	7.06a (0.27)	8.82a (0.12)
	4	8.29a (0.39)	6.78a (0.17)	7.89a (0.19)	8.44a (0.12)	7.27a (0.29)	7.36a (0.41)	8.13a (0.08)	8.23a (0.09)	7.92a (0.06)	7.09a (0.17)	9.18a (0.11)
	8	**8.57b*** (0.49)	6.88a (0.23)	7.85a (0.15)	8.73a (0.21)	7.34a (0.50)	7.48a (0.45)	8.00a (0.09)	8.14a (0.18)	7.83a (0.21)	7.28a (0.24)	9.45b (0.20)
	24	**8.95b*** (0.12)	8.05a (0.31)	8.40a (0.10)	**9.02a** (0.11)	8.35b (0.16)	8.27a (0.29)	8.35a (0.13)	8.35a (0.11)	8.31a (0.24)	7.92a (0.45)	**9.66b*** (0.05)
AX 1:3 β-glucan	0	7.47a (0.35)	7.24a (0.16)	7.62a (0.20)	8.20a (0.31)	7.45a (0.18)	7.24a (0.11)	7.92a (0.09)	8.06a (0.23)	7.74a (0.26)	7.18a (0.19)	8.82a (0.21)
	4	7.45a (0.26)	6.75a (0.20)	7.18a (0.28)	7.79a (0.15)	6.85a (0.15)	7.04a (0.22)	7.38a (0.19)	7.35a (0.15)	7.18a (0.11)	6.87a (0.09)	8.42a (0.09)
	8	7.82a (0.53)	6.88a (0.32)	7.60a (0.72)	7.83a (0.49)	7.06a (0.31)	7.30a (0.04)	7.88a (0.64)	7.98a (0.50)	7.89a (0.40)	7.43a (0.33)	8.84a (0.47)
	24	8.61a (0.43)	7.88a (0.08)	8.43a (0.60)	8.46a (0.36)	7.67a (0.20)	7.64a (0.25)	8.33a (0.53)	7.77a (0.56)	8.06a (0.38)	7.95a (0.18)	9.36a (0.38)
B glucan	0	7.21a (0.33)	6.93a (0.18)	7.33a (0.23)	8.04a (0.27)	7.29a (0.21)	7.05a (0.13)	7.65a (0.09)	7.89a (0.17)	7.46a (0.26)	6.99a (0.18)	8.59a (0.22)
	4	7.26a (0.36)	6.65a (0.22)	7.41a (0.54)	7.91a (0.35)	6.98a (0.40)	7.11a (0.41)	7.46a (0.39)	7.38a (0.37)	7.21a (0.26)	6.97a (0.29)	8.47a (0.35)
	8	7.54a (0.46)	7.07a (0.34)	7.78a (0.69)	8.01a (0.56)	7.38a (0.58)	7.36a (0.53)	7.72a (0.62)	7.42a (0.60)	7.56a (0.53)	7.41a (0.44)	8.73a (0.55)
	24	7.90a (0.24)	8.32a (0.30)	7.53a (0.41)	8.09 a (0.36)	7.51a (0.39)	7.20a (0.52)	7.58a (0.35)	6.86a (0.74)	7.20a (0.54)	8.11a (0.04)	9.10a (0.25)
